# Human tissue oxygen and strontium isotope values in North America: A data compilation and assessment for forensic geolocation

**DOI:** 10.1111/1556-4029.70030

**Published:** 2025-03-26

**Authors:** Kirsten A. Verostick, Alejandro Serna, Chris Stantis, Gabriel J. Bowen

**Affiliations:** ^1^ Department of Geology & Geophysics University of Utah Salt Lake City Utah USA; ^2^ Department of Archaeology University of York York UK; ^3^ División Arqueología Universidad Nacional de La Plata La Plata Argentina; ^4^ School of Anthropology, Political Science, and Sociology Southern Illinois University‐Carbondale Carbondale Illinois USA

**Keywords:** forensic anthropology, hair, human provenancing, oxygen isotopes, strontium isotopes, tooth enamel

## Abstract

The use of isotopic analysis for human mobility, geolocation, and forensic identification has become common over the past two decades, yet its effectiveness depends on the availability of well‐documented reference data. Many reference data exist in the literature, but the suitability of these data for forensic applications has not been critically assessed. Here, we compile oxygen and strontium isotope data for North American human tissues (hair, nails, bone, and tooth enamel). We review the geographic distribution of these data, evaluate their relationship with the predicted geographic variation, and assess potential sources of bias that may limit the comparability of different datasets in the compilation. A substantial number of data are available for some of these substrates and isotope systems, but in most cases, their geographic distribution is patchy with many areas under sampled. Except for hair Sr isotopes, a robust relationship occurs between human tissue values and modeled local environmental values, suggesting theoretically expected relationships between human tissues and local sources of O and Sr are expressed. These relationships are noisy; we identify several methodological differences that produce systematic biases across the compiled data. Based on these findings, we suggest interpreting forensic isotope data using currently published reference data is problematic. We propose the adoption of consistent metadata reporting and standardized laboratory protocols to enhance the utility of data gathered in future research as these practices could lead to measurable improvements in the strength of forensic interpretations derived from human tissue isotope data.

**Highlights**

- Over 3000 human tissue isotopic values from North America were published in the past two decades.
- Data conform to expected environmental and physiological mechanisms of isotope incorporation.
- Across studies, data show variations in metadata, geographic scope, lab methods, and tooth types.
- Standardizing methods, sampling, and reporting can improve data reliability and forensic utility.

## 1 INTRODUCTION

Oxygen (δ^18^O) and strontium (^87^Sr/^86^Sr) isotope values are now widely used in both forensic and archaeological contexts to address questions of human mobility, geolocation, and forensic identification. Isotopic analyses of O and Sr have proven invaluable as their abundance in human tissues derives from environmental sources people interact with and varies systematically in parallel with spatial isotopic patterns in the environment [1, 2, 3, 4, 5, 6]. The success of their application is rooted in how isotope values from the environment are incorporated into human tissues through food and water consumption. Coupled with physical, chemical, and biological processes that produce coherent isotopic variation in the environment, this results in predictable spatial variation in human tissue isotopic compositions that can be represented as isoscapes (i.e., quantitative predictive models of isotope variation in space and/or time represented in maps) [7, 8, 9].

Although high‐quality isoscapes exist for many environmental substrates relevant to humans (e.g., oxygen isotopes in drinking water [10, 11, 12] and strontium isotopes in food webs [7, 13]), robust application of isotope analysis in forensic contexts also requires reference data that document the isotopic composition of sample types used as evidence [14, 15, 16, 17, 18, 19]. Such data can be used directly as a benchmark for data from unknown samples [20, 21, 22, 23, 24] or compared with environmental isoscapes to calibrate and characterize the strength of the relationship between human tissue values and (generally more widely‐sampled) environmental values (see Ma et al. [17]). The latter approach capitalizes on the strong relationship expected between isotope values of human tissues and those of local environmental substrates and can be used to develop quantitative human tissue isoscape models for use in evaluating the origin of unknown samples.

The past two decades have seen a rapid growth in geolocation‐focused isotope research in North America. Researchers have focused on topics including taphonomic effects under specific burial conditions [25, 26, 27], isotope tissue–environment relationships [16, 28, 29], evaluation of multi‐isotope systems for geolocation across different regions [23, 24, 30, 31], and geolocation in different contexts (e.g., recording travel history [32, 33, 34, 35], undocumented border crossers [36], cold cases [37, 38, 39], and POW/MIA military personnel [18, 40]).

Despite the increasing generation of known‐origin isotopic data from this type of work, a synthesis and assessment of these data's potential as a reference dataset for human forensic geolocation is lacking. This study addresses this gap by compiling and critically evaluating human tissue δ^18^O and ^87^Sr/^86^Sr values from North America (Mexico, United States, and Canada). We report the scope and geographic distribution of existing data for two tissue types that have been a primary focus of research (hair/nail keratin and bone/tooth bioapatite) and consider several features of the datasets that may affect their comparability between publications or to newly generated data and the quality of the compiled dataset. We focus our analysis largely on hair keratin and tooth enamel bioapatite values, which comprise the vast majority of the available isotopic data.

We assess several factors that may influence dataset comparability and overall data quality, specifically (1) incomplete geographic life history data, (2) differences in laboratory methods, and (3) lack of standardization of tissue sampling (specifically, sampling of different dental elements). Our findings suggest that some of these factors may significantly influence the isotopic data, degrading the homogeneity of the data and reducing the strength of geolocation interpretations drawn from the compiled dataset. Based on these results, we recommend better practices for collecting and publishing known‐origin tissue isotope data, with the goal of enhancing their utility in forensic human identification.

## 2 MATERIALS AND METHODS

### 2.1 Literature review and isotopic data compilation

We reviewed and compiled data from modern human tissue samples in North America, with a focus on strontium and oxygen isotope values from human tissues including hair, nail, bone, and tooth. Multiple resources were used to find the information for this study, including several research databases (e.g., ScienceDirect and Wiley Online Library Journals), accessed via the University of Utah Library, and other platforms such as Google Scholar, Research Gate, and Pro‐Quest. The initial literature review was exhaustive, with an attempt to include any literature using, discussing, or publishing new strontium or oxygen isotopic data on human tissues. The bibliographies of the reviewed papers were also examined and cross‐referenced to further identify other relevant literature.

For the data compilation, we screened the compiled literature to identify studies publishing new data from samples where the location of tissue growth (hereafter “origin”) was reported (*n* = 27). These data were derived from journal articles, reports, theses, and dissertations. Not all the publications included the individual isotopic data values or complete metadata, and in these cases, we contacted investigators/researchers in an attempt to obtain the missing information. The isotope values and metadata were compiled and standardized as described below. We attempted to be inclusive, and data were compiled regardless of the completeness of the associated metadata, but we did not include studies where the integrity of the isotope values themselves was highly questionable or had known reported issues. The total database comprises >3000 isotopic data values (each δ^18^O value or ^87^Sr/^86^Sr ratio counted as a single value).

Sample data and metadata were compiled using a schema modeled on the IsoBank database [41]. A unique identifier (ID) was created for each site, individual, and dental or skeletal element analyzed. The original sample ID reported in the source publication was recorded when available, as well as the sex and age of the tissue donor. Because age reporting varied widely among studies, we categorized age as adult (18 years old or older) or nonadult rather than using a numerical value. The tissue type, dental or skeletal element sampled, and biomolecule analyzed were documented if reported.

Dental elements were categorized for analysis using a classification system based on crown formation periods as described by AlQahtani et al. [42]. Teeth were grouped into four categories: 0–4 years (first molars, central incisors), 0.5–6 years (lateral incisors, canines), 2.5–7 years (premolars, second molars), and 7.5–15 years (third molars). Some studies did not specify the exact dental element sampled, with reports ambiguously referring to a “molar” or leaving uncertainty about whether an incisor or canine was sampled. To account for these uncertainties, an “Unknown” group was included for samples that could not be assigned to a specific development stage.

Geographic locations (sites) were defined using information on city, state/province, country, latitude, and longitude, as provided. We classified each datum as either “known” or “assumed” origin. Known origin data are associated with specific geographic residence information, provided by or obtained on behalf of the tissue donor, covering the period of tissue growth. In contrast, residence history for assumed origin samples has been inferred indirectly by the researchers, for example, by assuming that the location of tissue growth is the same as that at which the tissue sample was collected. The assumption of origin is convenient in that it may eliminate the need for human subjects research oversight, but it adds uncertainty to data interpretation and may be increasingly tenuous as the time separation between sample collection and tissue growth becomes large (e.g., for teeth collected from individuals of advanced age).

When geographic data were not provided, GeoNames [43] was used to estimate the coordinates and elevations from the available geographic information. When only state/province or countries were provided, the centroid coordinates for the geographical unit of highest resolution were taken from GeoNames. When only coordinates were provided, reverse geocoding was used to provide city and state names. In some instances, the geographical information given was not adequate to derive coordinates (e.g., incorrect or ambiguous city name). These data were excluded from the analysis. Data with multiple origins (i.e., movement) included in the residential history were excluded from our data compilation and analyses unless all locations were in close geographic proximity (~20–30 km).

We compiled reported isotope values (δ^18^O and/or ^87^Sr/^86^Sr) and information on sample pretreatment protocols used, laboratory where analysis took place, instrumentation employed, and analytical standards used. Delta notation is used to express all oxygen isotopic values in per mil (‰), the standardized format for reporting isotopic ratios as deviations from a reference material. In contrast, strontium isotopes are reported as absolute ratios (^87^Sr/^86^Sr), reflecting the large absolute differences in Sr isotope ratios compared to light isotopes. When multiple analyses were reported for a single sample, these were designated by appending a code for the type of analysis after the Sample ID (O‐oxygen, Sr‐strontium, P‐phosphate, etc.). Oxygen isotope data reported on the VPDB reference scale were converted to VSMOW using the formula δ^18^O_VSMOW‐SLAP_ = (1.03092 × δ^18^O_VPDB_) + 30.92 [44] (conversions applied to three studies [18, 45, 46]). Enamel samples analyzed as phosphate (one study [47]) were converted to carbonate using Chenery et al.'s equation, δ^18^O_C_ = (δ^18^O_P_ + 9.6849)/1.0322, to facilitate comparison with carbonate values in the dataset. The dataset and code for analysis are held in a GitHub repository and openly available [48].

### 2.2 Methods‐data analysis

We conducted descriptive and exploratory analysis of data for all tissue types that were well represented in the data compilation (generally >100 samples from multiple studies), including hair oxygen, hair strontium, tooth enamel oxygen, and tooth enamel strontium. Data for other tissue types (e.g., bone, nail) were included in the compilation but not subject to statistical analysis due to the small number of available data. Data from two locations that fell outside of the mapped North American land area used in isoscape development (see below) were also excluded from all analyses. All statistical analyses were performed at a significance level of 0.05 using R version 4.3.1 [49]. The normality of data distributions were assessed using Shapiro–Wilk's test. Based on the results, Levene's test was applied for variance comparisons, and ANOVA was used for comparisons of group means.

We compared the tissue values with predicted local environmental values using the calRaster function in the assignR R package [17], which fits a weighted least squares regression relating the tissue values to environmental isoscape values at the locations of sample origin. Although we recognize that the environmental isoscapes do not fully represent the sources of O and Sr consumed by modern humans and are therefore not themselves appropriate for the interpretation of forensic data, we assume that they describe the first‐order geographic patterns in local sources of these elements that are taken into and may produce a geographically diagnostic isotope signature in human tissues. Through this comparison we: (1) assess the degree to which the hypothesized correlation with environmental substrates is reflected in the isotope values of human tissues, and (2) account for local influences on the tissue isotope values so that we can compare other characteristics of the datasets (discussed below).

For strontium, we used the global bioavailable strontium isoscape of Bataille et al. [7], accessed through the assignR package, as the source of the independent variable in our regressions. For oxygen, we developed a new isoscape of tap water isotope values for North America [50], produced using the method of Bowen et al. [8]. Tap water data were obtained from the Waterisotopes Database ([51] Query: Country = US, MX, CA, Type = Tap). In total, over 11,000 oxygen isotope values were pulled from the Waterisotopes Database, and after removing proprietary data and averaging multiple values from the same sites, 5275 values were used to create the North American tap water isoscape [8, 10, 11, 12, 28, 52, 53, 54, 55, 56, 57, 58, 59, 60, 61]. The tap water isoscape was generated by calculating the difference between tap water δ^18^O values and local mean annual precipitation δ^18^O values (from the global precipitation isoscape of Bowen [62], accessed through the assignR package), interpolating the difference values using ordinary kriging (R package gstat), and adding the interpolated difference values to the precipitation isoscape values. The new tap water isoscape is published and available in a GitHub repository via Zenodo [50].

**FIGURE 1:**
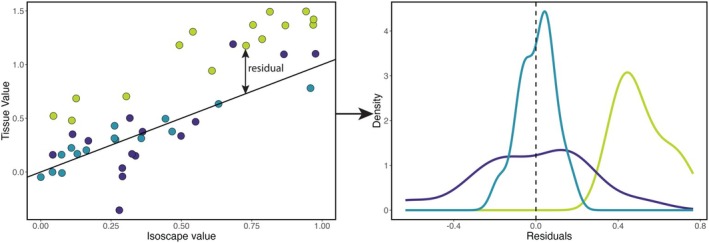
Schematic illustrating comparison of tissue–environment model residuals for different hypotheses. Data from the purple group give a broad distribution of residuals around the mean relationship as expected if data from assumed–origin samples exhibited a relatively weak tissue–environment relationship. Data from the green group exhibit residuals that are systematically offset from zero, as expected if differences in laboratory methods or tooth mineralization age bias these data relative to the average relationship for all samples. Data from the blue group demonstrate neither bias nor strong dispersion.

We analyzed residuals from the calRaster regression models for both O and Sr isotope values to identify systematic non‐geographic differences between data subsets. Residuals, defined as the difference between observed and calRaster regression model predicted values, reflect the extent to which individual data points deviate from the average relationship. If the characteristics of a particular subset of the data cause those values to deviate from the pattern defined by the rest of the data, this may be expressed in the anomalous distribution of residual values for that subset, schematically depicted in Figure 1.

We examined model residuals to test three hypotheses. First, we predicted that less precise geographic origin information would lead to a noisier relationship between environmental and tissue isotope values. This would be reflected in a broader distribution of residuals for assumed–origin (vs. known–origin) samples (e.g., the purple in Figure 1). Second, we predicted that biases in isotope data arising from the use of different pretreatment, analysis, and calibration methods would be reflected as offsets in the mean residual values for individual studies (e.g., the green data in Figure 1). Third, we predicted that differences in dietary and physiological factors affecting the incorporation of oxygen isotopes in tooth enamel would be reflected as systematic offsets in the mean residual values for data from teeth formed at different stages of childhood (e.g., the green data in Figure 1). In each of these cases, differences in the variance or mean values for residuals from different data subsets were assessed graphically and tested statistically to determine whether the hypothesis was supported.

Finally, we applied the QA (quality assessment) function in assignR to evaluate the potential impact of improved tooth enamel δ^18^O values standardization on the interpretation of data from unknown‐origin casework samples. Briefly, QA conducts an iterative split‐sample test in which a tissue isotope dataset is divided into calibration and testing sets. The former is used (via calRaster) to develop a tissue isoscape, and the latter is used to calculate metrics for the accuracy of geographic assignments made using that isoscape. The metric used here reports the fraction of testing samples that are correctly assigned (i.e., the reported origin is included within the assignment area) when using different posterior probability thresholds to split the study area (in this case, the North American continent) into regions that are likely and unlikely to contain the true site of origin. We conducted QA analyses using the compiled dataset as is and after applying a residual correction, wherein the mean calRaster residual value for data from each study or tooth mineralization age group was subtracted from the original (published) data (i.e., effectively removing any systematic bias between studies or age groups).

## 3 RESULTS AND DISCUSSION

### 3.1 Spatial distribution of compiled isotopic data

Descriptive statistics for the entire compiled dataset are presented in Table 1. Nail samples are underrepresented in published North American data and exhibit limited geographic distribution; therefore, these data were not included in statistical analyses. Similarly, bone apatite data, which are primarily available from the United States, were excluded due to their limited distribution.

**TABLE 1:** Descriptive Statistics of the entire complied dataset, arranged by Isotope, Country, and then tissue. Oxygen values reported in ‰ and ^87^Sr/^86^Sr values are reported as ratios.

Isotope	Country	Tissue	*n*	Mean	SD	Min	Max	Range
^87^Sr/^86^Sr	Canada	Tooth enamel	25	0.70938	0.00072	0.70810	0.71149	0.00339
	Mexico	Hair	101	0.70706	0.00180	0.70424	0.71613	0.01189
		Tooth enamel	155	0.70618	0.00105	0.70452	0.70866	0.00414
	U.S.	Bone	2	0.71017	0.00062	0.70973	0.71060	0.00087
		Nail (finger)	39	0.70982	0.00032	0.70921	0.71043	0.00122
		Hair	380	0.71021	0.00204	0.70660	0.71962	0.01302
		Tooth enamel	156	0.70983	0.00100	0.70600	0.71324	0.00724
δ^18^O	Canada	Hair	661	9.0	2.3	2.2	20.6	18.5
		Tooth enamel	60	22.8	1.5	19.6	25.5	5.9
		Nail (toe)	39	8.3	3.1	2.3	16.5	14.2
	Mexico	Hair	61	13.3	1.5	9.5	16.1	6.6
		Tooth enamel	156	25.3	1.4	22.1	28.1	6.0
	U.S.	Bone	248	19.7	4.1	10.9	27.8	16.9
		Hair	755	11.1	1.9	6.0	17.3	11.2
		Tooth enamel	333	25.1	2.3	18.0	30.6	12.6

**FIGURE 2:**
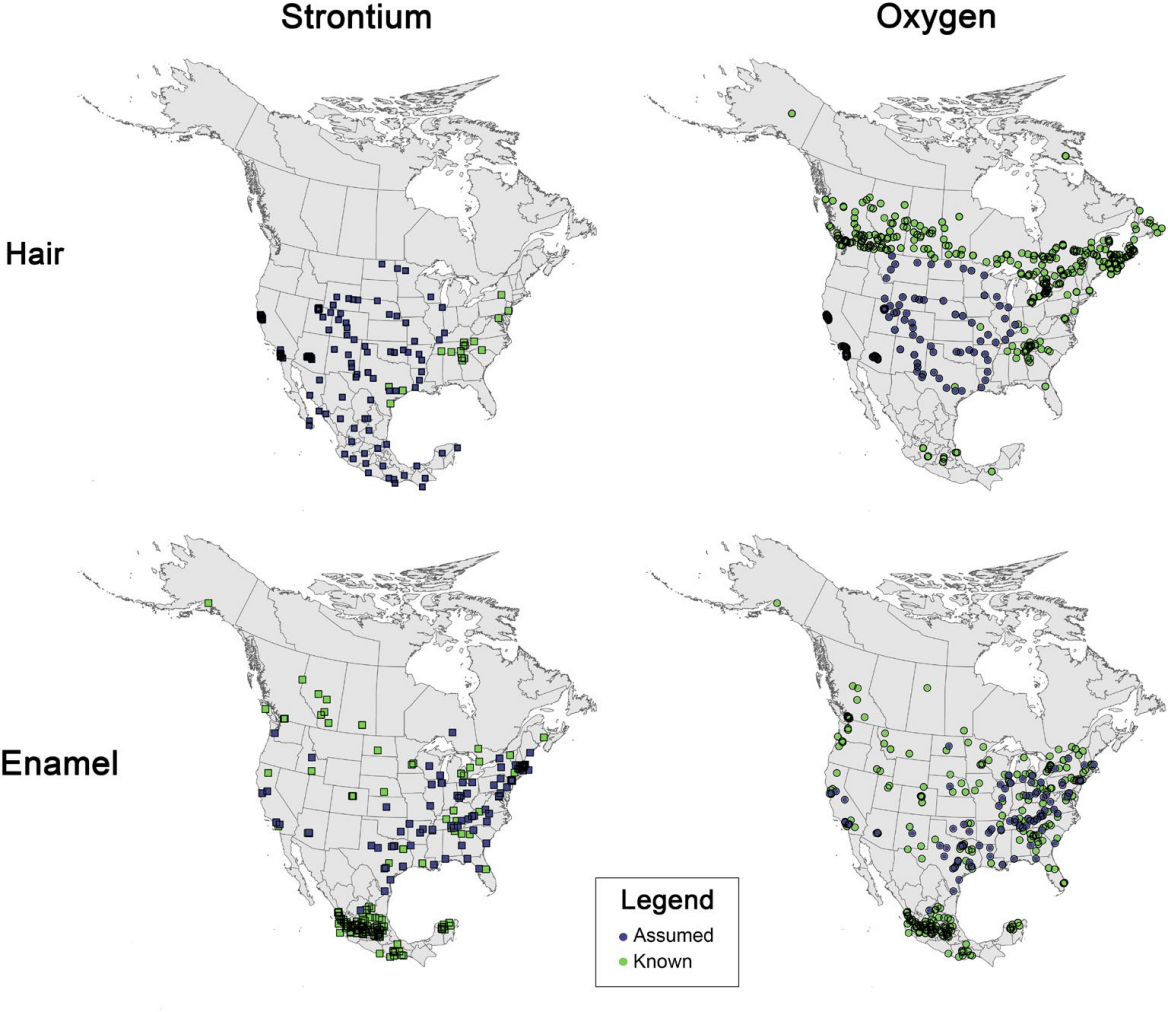
Maps showing the spatial distribution of published strontium and oxygen isotope values in North America for hair and tooth enamel. Assumed and known origin as described in Section 2.1.

The sampling density and spatial distribution for the two most widely measured sample types (hair and tooth enamel) are quite uneven across North America (Figure 2). For hair, oxygen isotope values are abundant and well distributed across the more densely populated parts of Canada [14, 33, 63, 64, 65] and are abundant but distributed more patchily throughout the United States The majority of the U.S. data are from assumed origin samples that were collected anonymously from barbershops, with reliable known origin data being limited to parts of the eastern United States [16, 35, 59, 66, 67, 68, 69]. Only a small number of hair δ^18^O values are available from central Mexico [28, 52].

Strontium isotope data are currently available only in the United States and Mexico. As for oxygen, U.S. strontium isotope values are dominated by assumed origin samples, with a smaller number of known origin samples collected in the eastern part of the country and a sampling gap across the northwestern United States [26, 67, 68, 70, 71]. Hair ^87^Sr/^86^Sr ratios from Mexico evenly represent most of the geographic area of the country but are obtained from assumed origin samples [72].

Tooth enamel δ^18^O values are inconsistently distributed across the continent, with very few samples from Canada [73] or northern Mexico. Samples are more evenly distributed across the United States, with denser sampling in the eastern part of the country and a blend of known‐ and assumed–origin samples [18, 45, 47, 74]. Samples from Mexico are predominantly from a small region of the country centered around Mexico City, with a small number of additional samples from the Yucatan and Oaxaca states [75]. The sampling distribution for tooth enamel ^87^Sr/^86^Sr ratios is similar to that for tooth enamel δ^18^O values, except for a slightly lower sample density across the United States [18, 45, 47, 75, 76].

Uneven sampling patterns expressed in the compiled dataset occurred for various reasons. Large‐scale studies in the United States and Canada have contributed numerous data from hair samples [14, 16, 69], but with distinct differences in scope and methodology. In Canada, a dataset with relatively even geographic coverage was built through a volunteer network that directly captured information on the donor's residence histories. In contrast, the U.S. study involved sample collection during driving trips conducted by the researchers. As a result, sampling is dense along certain linear routes, leaving large areas of the country unsampled. In addition, due to the rapid, low‐intervention strategy used in the U.S. transect collections, no personal or life history information was collected from donors, and the geographic origin of the samples could only be assumed.

Small‐scale studies, which often focus on specific regions, add valuable granularity to the dataset but present different challenges. For example, a dissertation project from the University of Tennessee's Forensic Anthropology Center contributed a large number of known‐origin hair data concentrated in a small region centered in eastern Tennessee [67]. Many such studies, typically constituting graduate theses or dissertations, rely on donated samples requiring special approvals for human subjects research, which often restricts them to smaller, regional sample pools. This regional focus contributes to the uneven and “patchy” sample distribution of the data compilation.

The contrasting methodologies and scopes of larger‐ and smaller‐scale studies reflect the logistical, financial, and regulatory factors shaping isotopic research with human tissues. Large projects, while costly and complex, offer broad spatial coverage but may lack detailed donor information. In contrast, small projects, though more limited in geographic reach, can yield data with precise origin details and rich contextual information valuable for mechanistic studies and characterization of isotopic variation within local populations.

### 3.2 Evaluating relationships between human tissues and environmental isoscapes

Environmental isoscapes (for bioavailable strontium and tap water oxygen) provide a first‐order prediction of spatial patterns that may be transferred to human tissues. In this section, we use comparisons between tissue isotope values and environmental isoscape values at the location of known or assumed tissue origin to test the expectation that isotopic variation in tissues will parallel that of the isoscape and to explore aspects of the dataset that may bias or degrade the tissue–environment relationships.

#### 3.2.1 Strontium in hair

**FIGURE 3:**
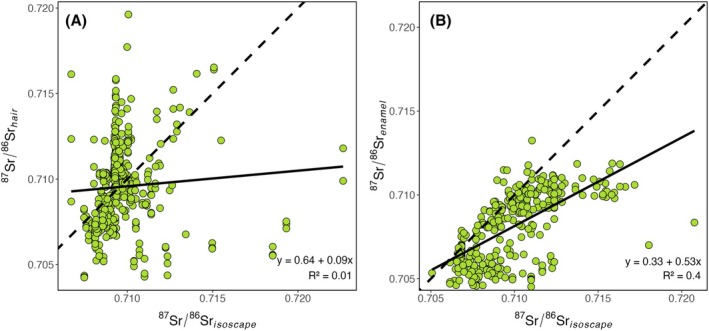
Tissue–environment relationships for ^87^Sr/^86^Sr ratios. (A) Strontium isotope ratios of hair regressed against modeled local bioavailable Sr (from the isoscape). (B) Strontium isotope ratios of tooth enamel regressed against modeled local bioavailable Sr (from the isoscape). Lines show the 1:1 relationship (dashed), and regression model (solid).

Strontium isotope ratios of human hair samples are uncorrelated with the isoscape‐modeled bioavailable ^87^Sr/^86^Sr ratios (Figure 3A), although both substrates exhibit a similar range of values (between ~0.705 and ~0.720). There is perhaps a weak suggestion of a covarying trend for samples with relatively low ^87^Sr/^86^Sr ratios (i.e., <0.710), but there is essentially no tendency for hair samples with higher (>0.710) values to be associated with origins where modeled bioavailable Sr isotope ratios are also high.

Previous work has shown that most strontium in keratinous human tissues is quite labile and can easily be assimilated from or exchanged with environmental substrates including bathing water and dust [25, 33, 69, 72, 77, 78]. This exogenous strontium, which is likely mixed with a small amount of strontium that is fixed during tissue growth, can be removed by sample cleaning [79]. All studies compiled here used a similar chloroform/methanol cleaning protocol, which is commonly employed to remove surficial oils from organic samples (e.g., [80]) and may be effective in removing adhered strontium [79]. In many cases, however, measurements made on keratin will sample strontium of mixed and/or uncertain origin. Although strontium isotopes in keratin may be useful in constraining very recent residence history in some cases [69], the complexity of strontium incorporation in hair and nail [77] suggests that geographic signatures and their interpretation may be messy, as seen in the simple tooth enamel–environment relationship presented here (see Figure 3A). Additional research on fundamental processes of Sr uptake in hair and the isotopic impact of variations in sample preparation and analysis may help standardize and inform the production and interpretation of such data.

#### 3.2.2 Strontium in tooth enamel

There is a moderately strong relationship (*R* ^2^ = 0.4) between measured tooth enamel ^87^Sr/^86^Sr ratios and those of the isoscape‐predicted bioavailable strontium (Figure 3B). This relationship is substantially stronger than the tooth enamel–environment relationship reported in an earlier assessment of modern humans from the USA [31], which may reflect the larger dataset compiled here and/or improvements in environmental isoscape models for strontium [7]. Unlike strontium in hair, however, the measured tooth enamel values spanned only ~50% of the range of bioavailable values predicted based on sample origins. The low slope of the relationship (0.53) is notable because many studies assume that local environmental strontium isotope ratios are transferred directly to biological tissues (thus predicting a 1:1 relationship). A substantial proportion of the tooth enamel data does cluster near the 1:1 line (see Figure 3B), and to some degree, the low regression slope can be attributed to the heterogeneity in the tissue–environment relationship and its impact on the regression fit using ordinary least squares.

That said, the slope is also influenced by a subset of data for which the environmental isoscape predictions are substantially higher (by ~0.005 or more) than the measured tooth values. The uncertainty of bioavailable Sr isoscape predictions is known to increase for higher ^87^Sr/^86^Sr ratios [7] and it is possible that prediction errors could degrade the quality of the tissue–environment relationship at some locations. A second likely explanation for the offset between predicted high bioavailable values and measured tooth values is that the human tissue samples may be integrating strontium from a range of sources, thus damping the expression of high ^87^Sr/^86^Sr ratios from local bioavailable strontium. This integration could occur due to multiple activities that are common within modern human societies, such as the consumption of nonlocal food and water (i.e., the modern “supermarket diet”) and travel beyond the local area [24, 31].

Another potential contributor to the heterogeneous or noisy tooth enamel–environment relationship could be the inclusion of assumed–origin samples for which the true location of residence during the time of tooth formation is not the same as the location of tissue sample collection. As described above, we hypothesized that a prevalence of errors in assumed origins would be reflected as greater residual variation around the best‐fit regression line (relative to the residual variation for known origin samples). In actuality, the dispersion of regression residuals is slightly lower for the assigned origin data (1σ = 0.0011) than for the known origin samples (1σ = 0.0016; Levene's test, *F*(1,331) = 24.8, *p* ≪ 0.001), which does not support our hypothesis (data not shown).

The number of assumed origin tooth samples is approximately half that of known origin (114 vs. 219) and assumed origin teeth were derived from only two studies (versus six studies for known origin teeth), meaning that other methodological differences may have inflated the residual variance in the known‐origin data (see below). Overall, however, our results suggest that errant assumptions about the location of origin of the teeth were not a major source of heterogeneity in the tooth enamel–environment relationship. Given this, the use of assumed origin samples in studies characterizing spatial patterns of tooth enamel ^87^Sr/^86^Sr ratios may not be particularly problematic, despite the high potential for individuals to have changed residence between the times of tooth mineralization and sample collection.

**FIGURE 4:**
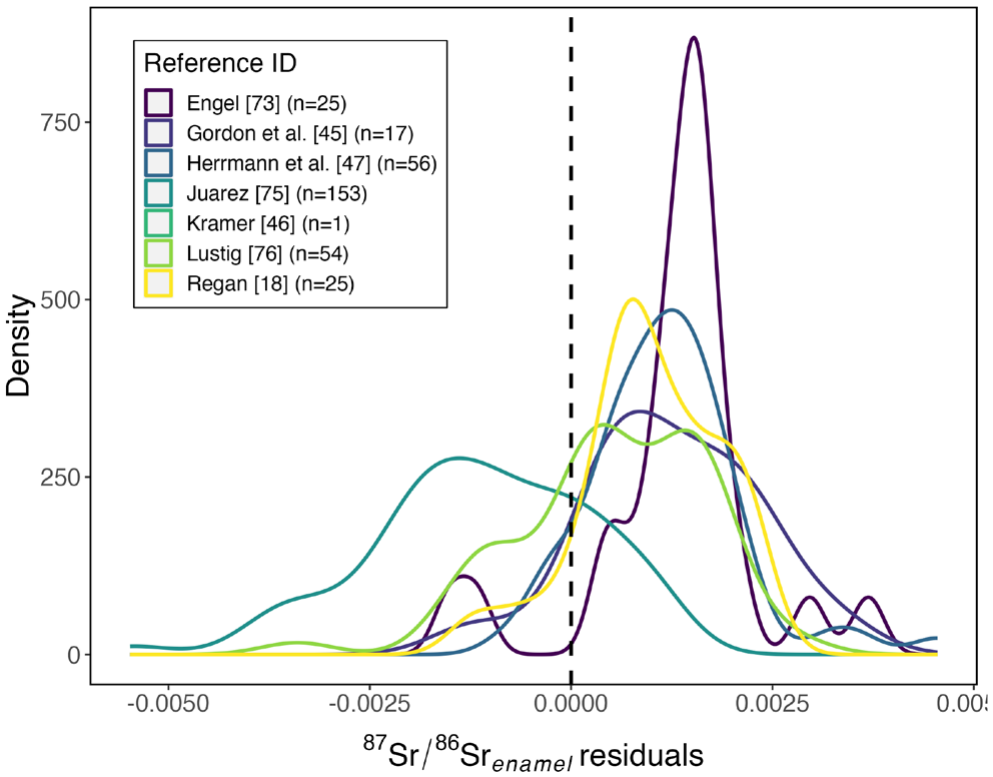
Tooth enamel strontium isoscape residual distributions presented by the study.

Methodological differences between studies may also contribute to residual variation in the tissue–environment relationships. We hypothesize that biases associated with different sample pretreatment, analysis, and data processing methods used in the different studies (Table 2) would lead to systematic offsets in the residual distributions. In this case, our analysis of data from the six studies does support the hypothesis (Figure 4; ANOVA; *F*(7, 325) = 36.8, *p* < 0.001). One study [75], in particular, yielded residuals that were shifted toward much lower values than the others. Although we are unable to conclusively identify methodological factors in that study that might bias isotope values, we note that it was one of two studies in which acids were used to pretreat the tooth enamel samples and that previous work has highlighted the potential for such treatments to affect measured ^87^Sr/^86^Sr ratios [81, 82].

**TABLE 2:** Pretreatment and preparatory protocols for tooth enamel strontium isotope analysis.

Reference/Study	Sampling	Cleaning/Pretreatment	Digestion	Chromatography	Instrumentation	Lab type	QC/SRM987
Engel* [73]	Powdered	5% acetic acid	5:1 mix of 24 N HF:16 N HNO_3_	Standard cation exchange, Ta gel	TIMS	Class 100	The typical precision associated with strontium isotopic analysis varies from 0.00001 to 0.00003 (2σ level)
Gordon et al. [45]	Powdered	Ashed 800°C	5 M HNO_3_, 2 M HNO_3_	0.32 M HNO_3_ **	MC‐ICP‐MS	Clean lab	Normalized ^86^Sr/^88^Sr value of 0.1194, ^87^Sr/^86^Sr = 0.710262 ± 0.000026 (2σ, *n* = 598)
Herrmann et al. [47]	Powdered	None reported	3 N HNO_3_, 3 N HNO_3_	Sr‐spec resin, 0.3 N H_3_PO_4_, Re filament, TaCl_5_	TIMS	Class 100	Not reported
Juarez [75]	Enamel chunks	0.5 N HCl etched, 0.1 N acetic acid	14 M HNO_3_, concentrated HCL, 3 M HNO_3_	Sr‐spec resin, super clean 2% HNO_3_	MC‐ICP‐MS	Not reported	Externally corrected to SRM987 using 0.710248, ^87^Sr/^86^Sr = 0.7102776 (±0.00003, 2σ)
Lustig [76]	Powdered	2%–3% NaOCl, 1 M acetic acid	1.5 N HCl, 3.5 N HNO_3_	Sr‐spec resin, 3.5 N HNO_3_, Re filament, Ta_2_O_5_ in 7% H_3_PO_4_	TIMS	Clean lab	Normalized ^88^Sr/^86^Sr to 8.375209, ^87^Sr/^86^Sr = 0.710243 ± 0.000006
Regan [18]	Powdered	None reported	50% HNO_3_, 6 N HCl, 1 N HBr, 3.5 N HNO_3_	EI Chrom Part#B100‐S resin, 2% HNO_3_	MC‐ICP‐MS	Class 1000	^87^Sr/^86^Sr corrected using ^86^Sr/^88^Sr = 0.1194. ^87^Sr/^86^Sr = 0.71025 (± −0.00004, 2σ)

* Pretreatment protocol not provided in dissertation.

** Nothing else is provided on chromatography protocol in the report.

Our results suggest that the lack of methodological standardization may be a significant factor limiting the comparability, and therefore the compilation, of tooth enamel strontium isotope datasets. Given that no universally accepted protocols for the preparation of tooth enamel and analysis of ^87^Sr/^86^Sr ratios exist, more experimental research toward the development of standard methods is warranted. In the meantime, clear and complete reporting of laboratory methods used in studies generating enamel Sr isotope data will be critical.

#### 3.2.3 Oxygen in hair

**FIGURE 5:**
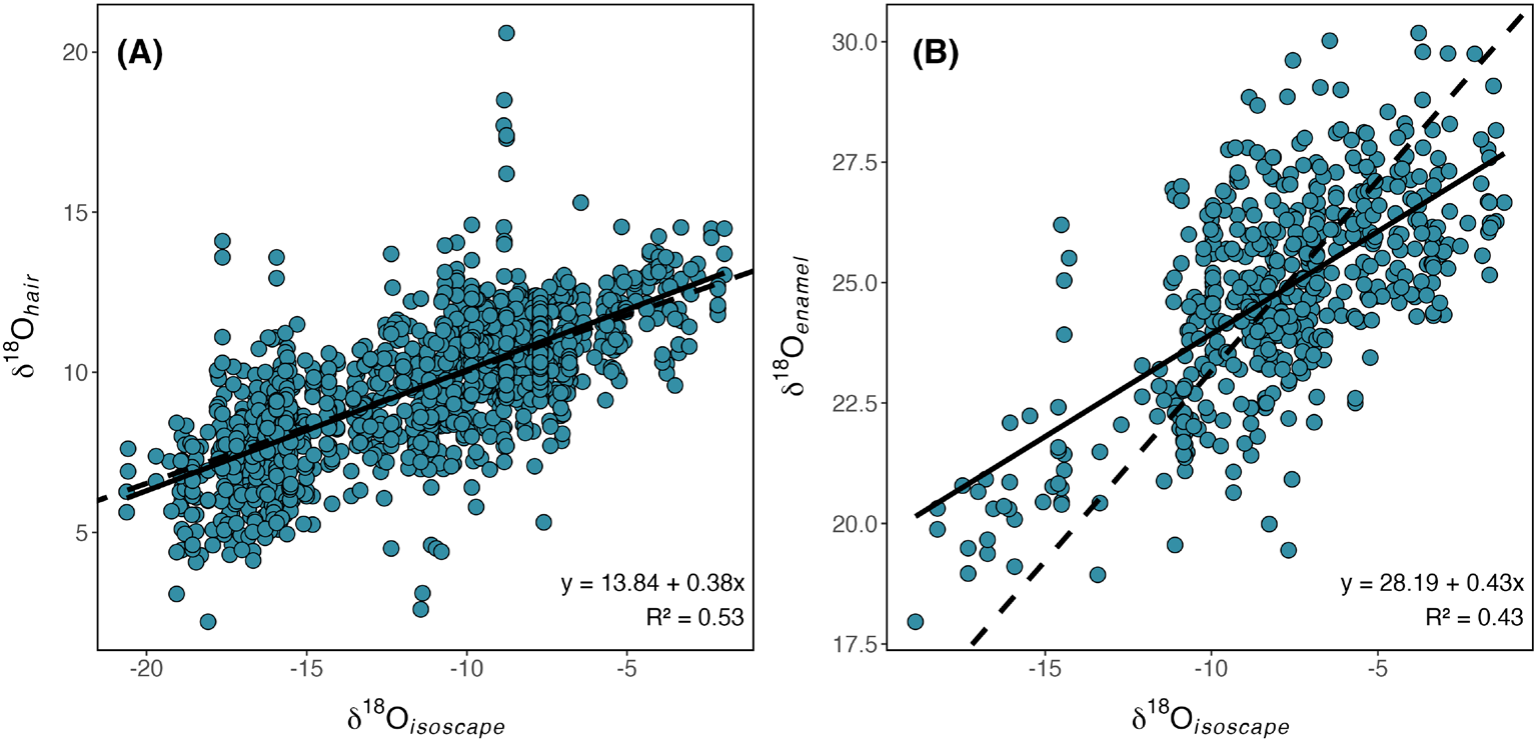
Tissue–environment relationships for δ^18^O values. (A) Oxygen isotope ratios of transformed hair regressed against modeled local tap water δ^18^O values. (B) Oxygen isotope ratios of tooth enamel regressed against modeled local tap water δ^18^O values. Dashed lines show the slopes of theoretical hair [16, 84] and body water model predictions [85], and solid lines show the regression model.

The development of widely accepted, certified, analytical reference materials for oxygen isotopes in organic compounds is still in process, and the studies compiled here use different reference materials developed in‐house to calibrate and report their data. Magozzi et al. [83] documented differences among these calibration scales and demonstrated that transforming keratin δ^18^O values onto a common reference frame improves comparability between data sets. We compared oxygen isotope data for the compiled dataset before and after transformation (conducted using the refTrans function in the assignR package) to a common scale (VSMOW; Table 3). The transformed hair data exhibited a slightly stronger relationship with isoscape‐modeled tap water (*R* ^2^ = 0.53, with a residual standard error (RSE) of 1.5‰, Figure 5A) than did the untransformed hair data (*R* ^2^ = 0.49, RSE = 1.7‰). Values for 23 samples from one study were excluded from the transformed dataset because their original calibration scale could not be determined; however, the difference in fit between transformed and untransformed data was identical if these samples were excluded from both datasets. This result suggests that variation in data calibration practices among laboratories may slightly degrade the strength of spatial patterns documented in the compiled human hair dataset, but the effect is modest. Nonetheless, given the positive result of this test, we conduct the rest of our analyses using the post‐transformation δ^18^O values to remove any effects related to the use of different laboratory reference materials.

**TABLE 3:** Calibration standards and reference scale (authoritative standard values against which data were reported) for δ^18^O values in each study. Terminology follows Magozzi et al. [83].

Reference	Calibration	Reference Scale	*n*
O'Brien and Wooller [35]	N/A		23
Chartrand and St‐Jean [14]	CAN_O_6	CAN_O_6	562
Mant et al. [64]	IAEA_O_2	IAEA_0_2	15
Ehleringer et al. [16]	OldUT_O_1	OldUT_O_1	210
Juarez et al. [52], Juarez et al. [28]	UT_O_2	IAEA_0_1	51
Saul [67]	UT_O_2	IAEA_0_1	44
Tipple et al. [69]	UT_O_2	IAEA_0_1	31
Tipple et al. [68], Tipple et al. [59]	UT_O_2	IAEA_0_1	325
Reynard et al. [66]	VSMOW_O	VSMOW_O	122
Ueda & Bell [65]	US_O_1	VSMOW_O	82

Oxygen isotope ratios in the transformed human hair dataset exhibit a robust correlation with the isoscape model tap water δ^18^O values (*R* ^2^ = 0.53, Figure 5A). Despite this strong relationship in the compiled dataset, the individual studies compiled here show that hair‐water *R* ^2^ values can approach or exceed 0.70 in some cases. The regression relationship between hair and water δ^18^O values has a slope much lower than 1, which, along with the moderate *R* ^2^, indicates other variables are likely influencing the tissue–isoscape relationship. The δ^18^O values of human body tissues are known to be influenced by a combination of oxygen from drinking water, food, and atmospheric O_2_ [16, 84]. In modern industrialized societies where most food products are obtained through centralized distribution systems, the δ^18^O values of both the food‐derived and atmosphere‐derived oxygen may be homogeneous, and local variation in δ^18^O values of hair may arise from isotopic differences in local drinking water alone. Models of oxygen isotope balance in the human body suggest that in such scenarios, hair should express ~35% of the regional variation in tap water δ^18^O values [16, 35, 84], similar to the relationship observed here across the North American dataset. Thus, unlike hair ^87^Sr/^86^Sr values, δ^18^O values in hair do appear to reflect the location of hair growth and broadly follow the predicted relationship with the environmental baseline.

We investigated the impact of differences in the quality of residential history information by comparing the distribution of tissue–environment residuals for the known and assumed origin data. Levene's test revealed a significant difference in residual variation between the groups (*F*(1,1450) = 19.0, *p* < 0.001), but like tooth ^87^Sr/^86^Sr ratios, the known origin group exhibited greater variance (1σ = 1.62) than the assumed origin group (1σ = 1.22). This suggests that the inclusion of assumed origin samples did not degrade the relationship between hair and tap water δ^18^O values or contribute strongly to the variation seen in the full dataset. The assumption underlying anonymous sample collection in many of the human hair studies was that individuals usually get their hair cut near the location of hair growth. Our result suggests that this assumption may be generally valid or, at least, examples where the assumption is violated are limited enough that they do not stand out above natural variation in the hair–water relationship for δ^18^O values.

#### 3.2.4 Oxygen in tooth enamel

The relationship between the measured δ^18^O values of tooth enamel carbonate (or carbonate‐equivalent values converted from measured phosphate) and the North American tap water isoscape is moderately strong (*R* ^2^ = 0.43, RSE = 1.6‰; Figure 5B). The regression slope (0.43) is similar to that observed for δ^18^O values of hair keratin and implies that variation in tooth enamel isotope values, like that of hair, is damped by contributions from sources other than local drinking water. In contrast, measured and modeled body water δ^18^O values for modern U.S. humans express approximately 79% of the spatial variation in tap water isotope values [85], which is much higher than that reflected by the least squares regression line slope fit to the North American hair dataset; however, it is not inconsistent with the general trend expressed by the data (Figure 5). We note that the slope of a reduced major axis regression is 0.65 (calculated as the ratio of standard deviations for the values on the *Y* and *X* axis), which more closely aligns with the value expected for body water. This regression would be appropriate if the uncertainty in drinking water δ^18^O values was of similar magnitude to that of tooth enamel. It is also possible that variation in the δ^18^O values of enamel mineral is damped slightly relative to body water due to the accumulation of metabolically derived water (containing isotopically invariant O from O_2_) within the environment of mineralization during amelogenesis.

We used the enamel–water residuals to test the hypothesis that some of the scatter in this relationship derives from the inclusion of samples of assumed geographic origin. Variance in the residuals for δ^18^O values of assumed origin samples (1σ = 1.53) was slightly larger than that of known origin samples (1σ = 1.46), but Levene's test indicated no significant difference (*F*(1, 539) = 0.03, *p* = 0.86). Assumed origin tooth samples represented ~25% of the compiled dataset and were generally referred to the location of birth by the original authors [46, 47]. Teeth mineralize early in life but as late as the early teenage years, and the data suggest that childhood relocation across environmental gradients was not common enough to compromise the tissue–environment relationship in δ^18^O values expressed in the dataset. This is consistent with demographic data from the United States indicating that long‐distance relocation is relatively rare even through early adulthood [86, 87].

Despite a long history of use in geolocation applications, no widely adopted standardized protocol for tooth enamel isotope sample preparation and analysis exists [88, 89, 90, 91, 92]. The studies providing the tooth enamel δ^18^O values compiled here used a wide range of laboratory protocols, with only two studies reporting identical methods (with the samples being prepared and analyzed in the same laboratory; Table 4). We hypothesized that differences in measured sample values resulting from contrasting sample pretreatment, analysis, and data processing methods would be expressed as systematic offsets in the distribution of tissue–environment model residuals for different studies.

**TABLE 4:** Pretreatment and preparatory protocols for tooth enamel oxygen isotope analysis.

Reference/Study	React temp (°C)	Oxidant %	Oxidant, plus time	M of acid	Acid, plus time	Baked	Standards used in Calibration	Preparation protocol source
Chesson et al. [40], Kramer [46]	26°C	3%	H_2_O_2_, 15 min	0.1 M	Acetic acid, 15 min	60°C overnight	NBS‐18, NBS‐19, LSVEC, USGS45	In Chesson et al. [40]
Gordon et al. [45]	Not reported	2%	NaOCl, 24 h	0.1 M	Acetic acid, 24 h	Unknown	NBS‐18, NBS‐19, LSVEC	In Gordon et al. [45]
Holobinko [74]	85°C	1.5%	NaOCl, 30 min	0.1 M	Acetic acid, 10 min	50°C, heat block,6 hours	NBS‐18, NBS‐19, LSVEC	Adapted by Lee‐Thorp [93, 94] and modification by Meier‐Augenstein [95]
Juarez [75]	90°C	2%–3%	NaOCl, 24 h	1 M	Buffered Acetic acid, 12 h	60°C, oven, one hour	NBS‐19, Carrera Marble	In Juarez [75]
Regan [18]	90°C	30%	H_2_O_2_, 24 h	0.1 M	Acetic acid, 20 min	No	NBS‐19	In Regan [18]
Ueda and Bell [29]	72°C	50%	NaOCl, 45–60 min	0.1 M	Acetic acid, 5–15 min	No	NBS‐18, NBS‐19, Cavendish Marble	Lee‐Thorp et al. [93]

*Note*: Herrmann et al. [47] samples were prepared and run for phosphate analysis. Phosphate analysis uses a very different chemical pretreatment protocol and standards. The phosphate values were converted (formula in Section 2.1 above) for comparison and use in Figure 6.

**FIGURE 6:**
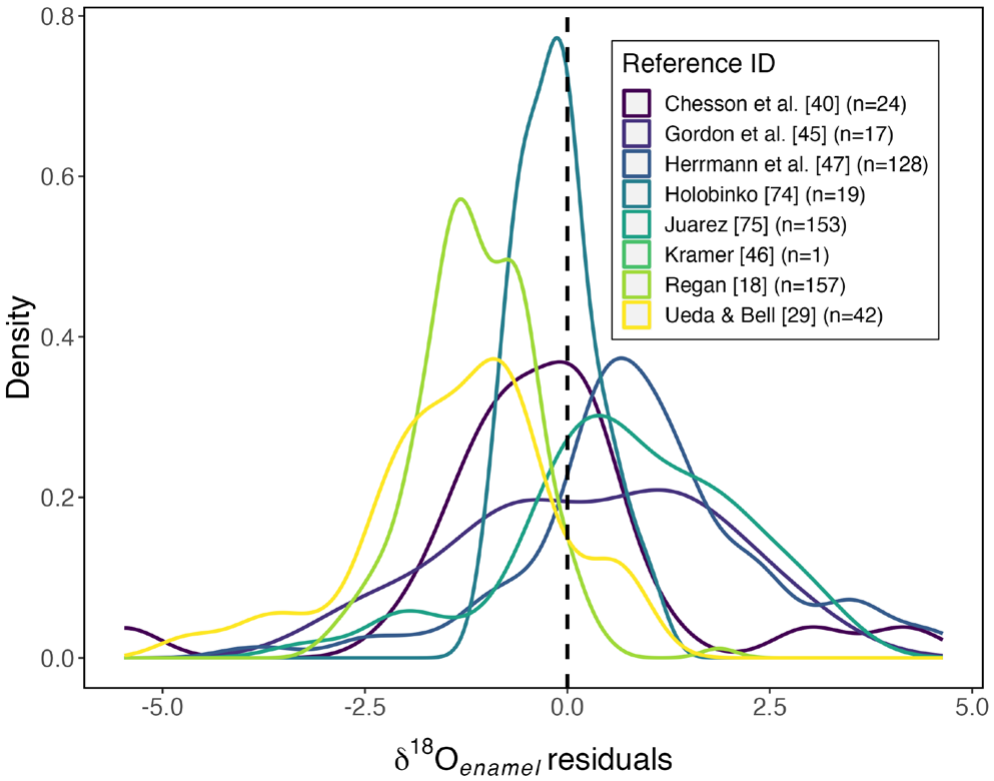
Tooth enamel oxygen isoscape residual distributions presented by the study.

This hypothesis is strongly supported, with the residual mean varying by ~3‰ among studies (Figure 6; ANOVA, *F*(7,533) = 42.8, *p* ≪ 0.001). Although the methodological variation among studies is too complex to isolate specific factors contributing to this result, we note that two of the studies [45, 75] with the most positive mean residuals reported using the most aggressive (longest and/or highest concentration) acetic acid treatments. Methodological research has shown that acetic acid treatment is associated with a substantial positive shift in measured enamel carbonate δ^18^O values [88, 90], and this treatment may contribute to the residual bias in the two high‐residual studies.

**FIGURE 7:**
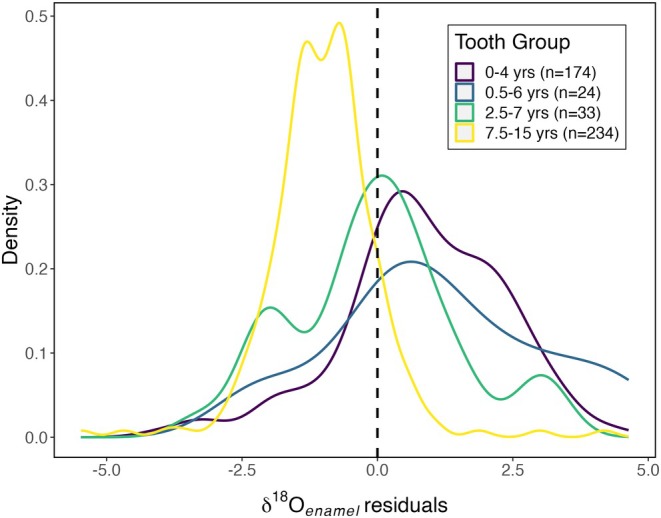
Tooth enamel oxygen isoscape residual distributions presented by age range of tooth formation.

A final source of potential variation examined in the tissue–environment relationship for δ^18^O values is the timing of tooth development and mineralization. The studies compiled here sampled a range of different tooth types and varied in the level of standardization of which teeth were sampled. Tooth enamel begins forming in utero and continues throughout childhood into the mid‐teens. Throughout this period there are also characteristic changes in diet (e.g., weaning) and physiology (e.g., progressive reduction of metabolic rate relative to body mass and body water turnover). These changes will affect the body water isotope balance [96], and, in general, are expected to produce a progressive reduction in body water (and thus newly mineralized tooth enamel) δ^18^O values as an individual ages. We hypothesized this influence would be expressed as a difference in the mean tissue–environment residuals for teeth formed at different stages of childhood. The residual distributions for teeth formed at different ages (Figure 7) support this hypothesis, with significant differences between the mean residuals for different groups (ANOVA, *F*(3,461) = 71.6, *p* ≪ 0.001). Moreover, the mean residual value generally declines with increasing age of tooth mineralization, consistent with predictions for the influence of weaning and physiological development [96, 97, 98, 99].

### 3.3 Implications of data standardization for forensic geolocation

The results presented above suggest that the standardization of several factors, including laboratory methodologies for sample preparation and analysis, and the dental elements sampled, are likely to improve the comparability of known/assumed–origin isotope data and the fidelity of the geographic patterns they document. To explore the effect of such standardization, we first bias‐corrected the compiled tooth δ^18^O values by subtracting the mean residual values observed for each study (see Figure 6) or tooth group (see Figure 7). Using both the original (as published) and bias‐corrected data, we then analyzed the proportion of the samples correctly assigned to their known/assumed origin using the assignR QA function. This metric represents the geographic specificity of the interpretations that can be drawn from the isotopic data, which we would expect to increase where the bias‐corrected data record more coherent and less heterogeneous or noisy geographic isotopic patterns.

**FIGURE 8:**
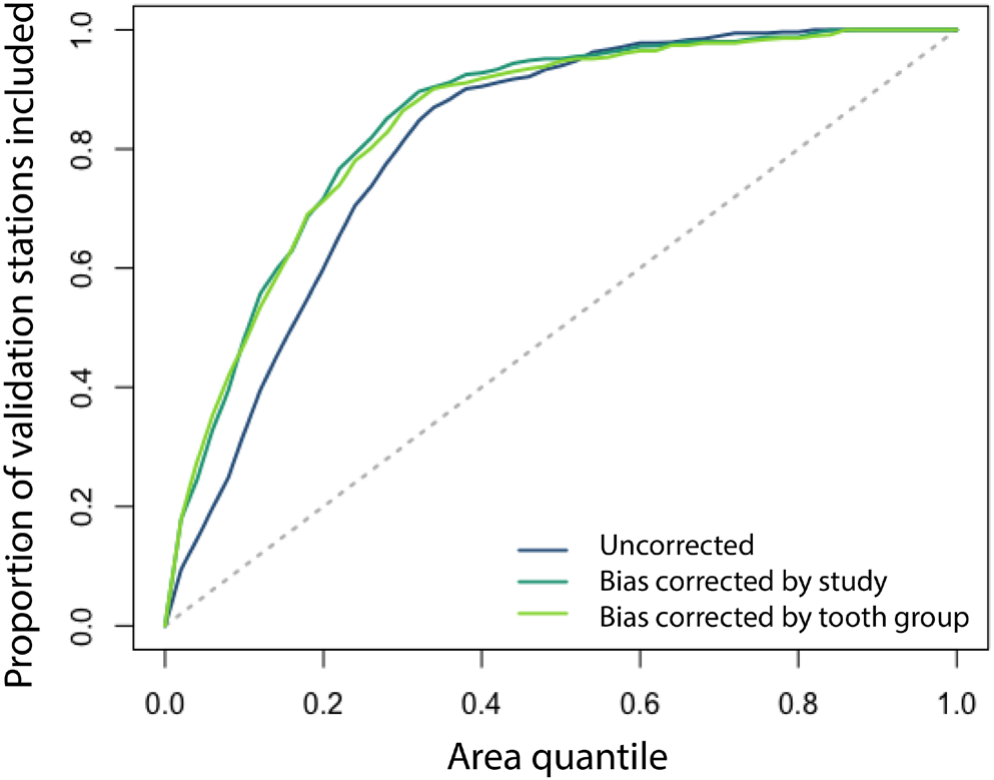
QA plot showing the proportion of validation samples correctly assigned to their location of origin based on δ^18^O values as a function of the assignment area (fraction of the total study area). The dashed line represents random assignments, as expected if the isotope data provide no useful geographic information.

Results show a ~20% improvement in geographic specificity when using tooth enamel δ^18^O values that are bias‐corrected by either the study or tooth development age group (Figure 8). The improvement is strongest when samples are assigned to a relatively small region of the study area (small “area quantile” values). For example, assignments that targeted the most likely 10% of the North American land area based on the isotopic evidence correctly predicted the origin of ~35% of the samples using the dataset without bias correction; for either bias‐corrected dataset the level of accuracy for predictions increased to >50%. Conversely, an investigator who wanted to identify the origin of an unknown sample with 80% confidence could expect to be able to eliminate ~70% of the North American continent, on average, based on the uncorrected data compilation, increasing to ~75% using the bias‐corrected data.

These results are illustrative, and given the inconsistencies inherent in the data compilation, we do not recommend the use of the fully compiled dataset in forensic geolocation efforts. This is particularly true for tooth oxygen and strontium isotopes, where we found strong systematic biases between data subsets; the results for hair oxygen isotopes suggest that the combined dataset may be useful after accounting for differences in calibration. The results also suggest that real gains in the accuracy and specificity of isotope‐based geolocation may be achieved through improved standardization of reference datasets. Moreover, they emphasize the importance of comprehensive reporting of study methods in publications that include known‐origin data: without this information, it is impossible to assess the degree to which methodological factors may compromise the use of published data as reference samples in forensic casework.

Although inconsistencies in the compiled data may limit their utility in forensic casework, practitioners may be able to use subsets, for example from specific studies, as reference data. Our results suggest that robust use of the data in this way will require careful assessment to ensure that the sampling and laboratory practices used to produce the reference data match those applied to the unknown materials and minimize the potential for these factors to bias forensic interpretations. Our work highlights specific factors that are most likely to induce bias for different tissues and isotope systems, which can guide scientists in the responsible use of the data compiled in this study.

## 4 CONCLUSIONS

Reference isotope data from human tissues provide an essential benchmark for interpretations in human forensics, including applications involving questions of geographic provenance. Our synthesis shows that many potential reference data from North America have been published in the literature and that in most cases these data reflect anticipated patterns of geographic variation and correlation with oxygen and strontium isotope values of the local environment. That said, large geographic sampling gaps exist, and heterogeneity in sample types and study methods emerge as important sources of heterogeneity or “noise” that reduces the accuracy and specificity of isotope‐based interpretations of geographic origin. Improving the quality and standardization of reference datasets will be important for advancing the utility and acceptance of isotope analysis as a tool for human forensic identification.

We offer several suggestions for better practices that will improve the comparability and facilitate the use of isotopic reference data. Researchers should strive to report comprehensive metadata documenting their samples and laboratory protocols. These include information on sample origin (and how that origin was determined), dental/skeletal element sampled, chemical and physical pretreatment of samples, analytical instrumentation and protocols, and correction and calibration of raw isotope values. Although our work suggests that samples collected without obtaining detailed life/residential histories of tissue donors (“assumed origin”) have not significantly compromised the quality of the existing North American tissue datasets, we do not endorse this as a best practice for data [17, 23, 24, 30, 100, 101, 102] that may be used in forensic casework where the validity of reference datasets may be called into question.

Methodological research continues to improve our understanding of the impact of different sample preparation and analysis protocols on isotope measurements of human tissues and yield information to support the development of better practices [81, 88]. Agreement on and adoption of standardized laboratory methods will be another critical step in the process of generating isotope data that supports robust interpretation, allowing isotope data to rise to standards imposed on forensic evidence (e.g., Daubert v. Merrell Dow Pharmaceuticals, Inc., 509 U.S. 579 [103]).

Finally, recognition of isotopic differences among related body tissues (i.e., different teeth within the dentition) is critical and demands careful documentation and reporting of sample information to ensure like‐to‐like comparisons with reference samples. The observation that data from the diverse and heterogeneous studies compiled here qualitatively match theoretical expectations for changes in body water δ^18^O values during childhood is encouraging and suggests that further research could develop a general model for the interpretation of data from different tooth types. In the near term, however, it implies that forensic interpretations of tooth enamel δ^18^O values should be based on comparisons with reference data representing the same tooth position or, at minimum, developmental stage.

## FUNDING INFORMATION

Support for this research was provided by a grant (#4666) from the U.S. Department of Defense and the Henry M Jackson Foundation for the Advancement of Military Medicine (https://hjf.org). The funders conducted a compliance review of the manuscript prior to submission but otherwise had no role in study design, data collection and analysis, decision to publish, or preparation of the manuscript.

## CONFLICT OF INTEREST STATEMENT

The authors have no conflicts of interest to report.

## Data Availability

Data and code are held in GitHub and can be found at: https://doi.org/10.5281/zenodo.14902878.
